# Seismic Exploration Wireless Sensor System Based on Wi-Fi and LTE

**DOI:** 10.3390/s20041018

**Published:** 2020-02-13

**Authors:** Zhiyuan Yin, Yan Zhou, Yongxin Li

**Affiliations:** School of Mechanical Engineering, Nanjing University of Science and Technology, Nanjing 210094, China; 312012210@njust.edu.cn (Z.Y.); liyongxin_a@njust.edu.cn (Y.L.)

**Keywords:** wireless seismic networking, seismic sensor array, dual-layer network, reliability mechanisms

## Abstract

In present seismic exploration wireless sensor systems with large acquisition channels, it is difficult to achieve a high data rate, high reliability and long distance in wireless data transmission simultaneously. In this paper, a wireless seismic exploration system using a dual-layer network is proposed. The dual-layer network is designed based on Wi-Fi and LTE, so that long-distance high-rate seismic data transmission with a high reliability can be achieved. In the proposed system, the sensor array is composed of two kinds of nodes, the gateway node and the collecting node. Based on the proposed nodes, collecting node positioning, seismic data acquisition, seismic local data storage and quasi real-time remote seismic data transmission can be realized. Reliability mechanisms have been put forward to deal with the exceptions. An experiment was carried out to test the data transmission efficiency of the proposed system. The results show that the seismic exploration wireless sensor system with a dual-layer network structure can achieve quasi real-time remote seismic data transmission with no packet loss.

## 1. Introduction

Seismic exploration is an important geophysical exploration method. Wireless seismic exploration systems with large acquisition channels (nodes) are the main forms of present seismic exploration equipment. Various wireless seismic exploration systems have been developed successfully and have been used in seismic exploration. The UNITE system, produced by Sercel, and the RT2 system, produced by Wireless Seismic, are typical wireless seismic exploration systems.

The UNITE system [1,2,3] is a wireless seismic exploration system based on Wi-Fi. It is mainly composed of a recording truck (the central control station), remote acquisition units (RAUs) and cell access nodes (CANs). CAN is the data exchange node. A star-mesh structure is used in the system. The CAN forms a star network with the RAUs. CANs form a mesh network with the recording truck. The communication between the RAUs and a CAN is based on 802.11b. CANs communicate with the recording truck based on 5.8 GHz Wi-Fi. Based on the above network topology, RAUs can collect, store and transmit seismic data to the recording truck according to its instructions. The data transmission of UNITE works in unlicensed bands, so the communication can be used without authorization. The actual short-distance transmission rate, based on 802.11b, is normally less than 1 Mbps in outdoor line-of-sight environments. In order to achieve a transmission distance of 1000 m without a significant reduction in transmission rate, each CAN needs to use a high-power antenna kit, which results in an increase in power consumption.

The RT2 system [4,5,6,7] is composed of a center record system (CRS), cross-station line interface units (LIUs) and wireless remote units (WRUs). A dual-layer multi-hop chain topology structure is used in the RT2 system. The lower layer is a multi-hop chain topological network, a line composed of multiple WRUs and one LIU. The upper layer is a multi-hop chain topological network consisting of the CRS and multiple LIUs. The communication of the lower layer is in the 2.4 GHz band and the communication of the upper layer is in the 5.8 GHz band. Based on the above network topology, WRUs can collect, store and transmit seismic data to CRS. The multi-hop chain topology network makes the system able to achieve a large acquisition channel with a limited number of LIUs. However, congestion is probable near the LIU, since the traffic load rises with the number of hops, which can decline the data rate sharply. Failure of a WRU will probably lead to separation of the downstream WRUs. Failure of a LIU can lead to the separation of a line.

The bucket brigade sensor array [8] proposed by Wireless Seismic has a similar structure to RT2. The main difference is that the even-numbered collecting nodes and odd-numbered collecting nodes form two multi-hop chain networks with the cross-station for the uplink communication of the lower layer. Two uplink multi-hop chain networks in one line reduce the number of hops, so the data transmission is more efficient in the lower layer. To maintain the data transmission of the downstream nodes in the line, network healing was designed for the communication failure of a collecting node. However, when the failure of continuous adjacent collecting nodes or a cross-station occurs, the separation is still probable. 

In addition to the above typical systems, some researchers proposed wireless seismic exploration systems with different features.

Savazzi et al. proposed a high-density seismic exploration system using ultra-wide band (UWB) and long-range Wi-Fi [9,10]. In the system, the node array is divided into several subnetworks. A subnetwork includes a gateway node, dozens of cluster-head nodes and hundreds of leaf nodes. The subnetwork is made up of cluster-mesh architecture based on UWB. The lossy data compression is used to reduce the traffic load and enhance the efficiency of data transmission.

Reddy et al. proposed a seismic exploration system using 802.11af in television white space (TVWS) bands [11]. A dual-layer star topology structure is used in the system. The range of communication is 2 km. If the number of nodes in a star network is limited, the data transmission efficiency is high. However, collecting nodes require 1m antennas and gateway nodes require 3m antennas.

The above typical seismic exploration systems adopt dual-layer network architecture; Wi-Fi or UWB high-speed transmission technology is used in the lower layer network, and high-power Wi-Fi technology is used in the upper layer network to realize the on-site transmission of exploration data. A disadvantage is that the increase in transmission distance leads to sacrificing the transmission rate or increasing the power consumption.

Low-power wide-area (LPWA) wireless technologies are effective long-distance wireless data transmission technologies [12]. LoRa and NB-IoT are typical LPWA technologies. Their advantages are long transmission distances and strong penetration. However, because of the low transmission rate, LPWA technologies are not suitable for a seismic exploration system with a large amount of data. 

Long term evolution (LTE) mobile communication technology is also an effective long-distance wireless data transmission technology [13]. LTE technology can achieve long-distance high-rate transmission. However, for a large-scale observation array, such as the one in [13], the arrangement of multiple 4G masts is required, leading to a very high labor cost.

Seismic data wireless transmission systems based on Wi-Fi [14,15,16,17], 802.15.4 [18,19,20,21,22] or other communication protocols [23,24,25,26] for other fields, such as seismic monitoring, are designed with a small-scale sensor array, a low sampling rate or partial data transmission, which are also not suitable for large-scale seismic exploration systems.

In summary, for present wireless seismic exploration systems based on a single wireless communication technology, it is difficult to achieve high rate, long distance and high reliability in data transmission simultaneously. Data transmission efficiency has become the technique’s bottleneck. To break through this bottleneck, an effective way to combine short-distance high-rate transmission technology with long-distance high-rate transmission technology is needed.

A wireless seismic exploration system based on Wi-Fi and LTE communication technology is proposed in this paper. A dual-layer network is designed for the system. The lower layer network is a star network based on Wi-Fi and the upper layer network is an LTE network. The lower layer network includes collecting nodes and a gateway node. The communication between the collecting nodes and the gateway node is short-distance Wi-Fi communication of a high rate and high reliability. The gateway node has the functions of data aggregation and forwarding. It aggregates data from collecting nodes and forwards data to a seismic workstation through the LTE network. 

In the seismic exploration area with no mobile communication network, a seismic workstation equipped with a private LTE base station can work with several hundred collecting nodes using the proposed network. There is only one hop between the collecting node and the gateway node, and high-rate Wi-Fi is used, making the data transmission efficient in the lower layer. Combining the LTE network in the upper layer with efficient data transmission in the lower layer, long-distance high-rate seismic exploration data transmission is achieved. With the particular designs of the proposed nodes, quasi real-time remote seismic exploration data transmission with a high reliability can be realized.

The rest of the paper is organized as follows: the architecture of the seismic wireless sensor system is presented in the next section, Section 3 introduces the designs of the proposed collecting node and gateway node, and Section 4 introduces the reliability mechanisms for exceptions. In Section 5, the data acquisition and transmission experiment results are provided to show the performance of the proposed system. Section 6 concludes the paper.

## 2. Architecture of the System

### 2.1. Design Idea of Seismic Wireless Sensor System

To achieve efficient data gathering in seismic exploration, long-distance high-rate wireless data transmission with a high reliability from the seismic sensor array to the workstation is required. Zigbee, UWB or Wi-Fi can hardly achieve long-distance high-rate transmission. LTE mobile communication technology, however, can achieve this type of transmission. Usually there is no mobile communication network in the seismic exploration area, so the private base station needs to be well-equipped. Due to the limitations on concurrent users, using LTE technology to transfer data directly from node to workstation will limit the scale of the sensor array. Integrating short-distance high-rate wireless data transmission with LTE is an effective solution.

The main design idea is that the transmission is based on a dual-layer network, and the sensor array is divided into subnetworks. Star topology is used in the subnetworks. Each subnetwork is composed of a gateway node and between five and 20 collecting nodes. The number of collecting nodes in a subnetwork is flexible to adapt to the terrain of the exploration area. Gateway nodes communicate with collecting nodes in the lower layer and then communicate with the seismic workstation in the upper layer. Wi-Fi is used in the lower layer to achieve short-distance high-rate data transmission. LTE is used in the upper layer to achieve long-distance high-rate data transmission. In the dual-layer network, the maximum number of collecting nodes can be between five and 20 times the limitations on LTE concurrent users.

### 2.2. Overall Structure

**Figure 1:**
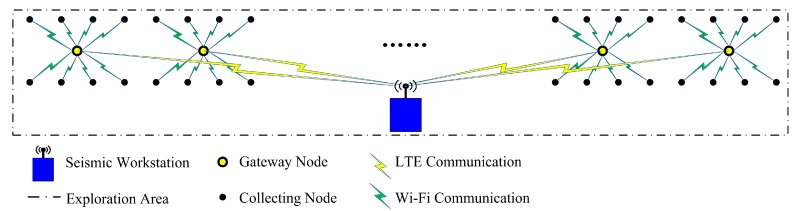
System structure of proposed seismic wireless sensor system.

The typical system structure of the proposed seismic wireless sensor system is shown in Figure 1.

In Figure 1, the collecting nodes are arranged into two long lines. The in-line offset is 10 m, the cross-line offset is 20 m, and a sensor array over a rectangular lattice is formed. The sensor array is composed of 400 collecting nodes, with 200 collecting nodes in each line. Eight collecting nodes and a gateway node form a subnetwork, and a gateway node is placed in the center of each subnetwork. Thus, the gateway nodes are also arranged into a long line. There are 50 subnetworks in the sensor array. The seismic workstation and the gateway nodes form the upper layer network. The above parameters of the sensor array are adjustable to fit different field operation requirements.

In seismic exploration, the basic function of a collecting node is to record the required seismic data. A collecting node communicates with a gateway node using short-distance Wi-Fi to receive instructions and to upload recorded data.

Each gateway node is the connecting link between the seismic workstation and the collecting nodes in the corresponding subnetwork. A gateway node joins the upper layer network to communicate with the workstation using LTE. It is the center node of a star topology subnetwork, namely the Wi-Fi access point (AP). In the upper layer network, each gateway node receives instructions from the workstation, implements corresponding actions and forwards aggregated data to the workstation. In each lower layer subnetwork, the gateway node sends instructions to the collecting nodes and also receives data packets from them. The gateway node is the data aggregation node and repeater.

The seismic workstation is normally a recording truck, and it is equipped with a private LTE base station in the proposed system. The main functions of the workstation include sending instructions, receiving data, storing data, managing the database and showing the states of nodes. The workstation forms a star network with the gateway nodes using LTE. The communication range of a private LTE base station can be several kilometers, and the base station can support 100 concurrent users. 

Based on the proposed structure, the system can achieve wide-area seismic exploration. The dual-layer network based on LTE and Wi-Fi can achieve quasi real-time long-distance high-rate seismic data transmission.

## 3. Node Design

### 3.1. Collecting Node

**Figure 2:**
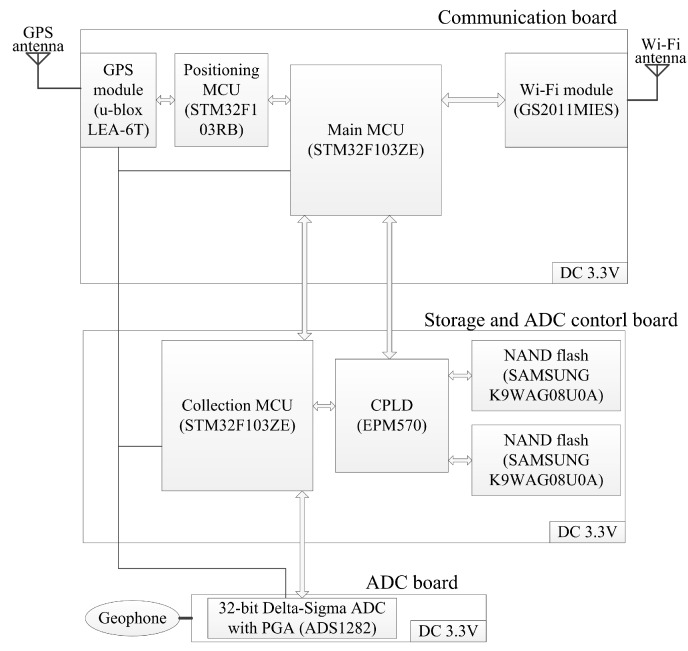
Schematic overview of collecting node structure.

The collecting node should achieve functions including synchronous seismic data acquisition, data storage, node positioning and Wi-Fi communication. The collecting node is mainly composed of three boards. Figure 2 shows a schematic overview of the collecting node structure.

The communication board mainly achieves Wi-Fi communication and node positioning. The storage and ADC control board is designed for seismic data local storage and the ADC board control. Analog-to-digital converter (ADC) board operates signal acquisition and conversion.

The main microcontroller unit (MCU) decides the main actions of the node according to the instructions from GS2011MIES, gets the required data from other modules, and sends the required data by GS2011MIES. GS2011MIES provides 802.11b/g/n radio, with the peak rate of 72 Mbps for a 20 MHz channel in 802.11n 1x1 single stream mode, and connects to the main MCU by SDIO. An omnidirectional circular rod Wi-Fi antenna is used. The antenna length is less than 0.3 m, and the gain is between 3 dBi and 6 dBi in different circumstances. The positioning MCU achieves the configuration of LEA-6T and the differential positioning with LEA-6T, so that the main MCU is not continuously occupied by these two actions. 

The collection MCU sends commands to control the ADC board, reads the acquired seismic data from the ADC board and stores the acquired data in the NAND flashes. The NAND flashes provide 2 GB storage.

Moving coil geophone is linked to the ADC board. The typical geophone front-end circuit of ADS1282 is used on the ADC board [27]. The sampling rate of ADS1282 is selectable and up to 4000 sample per second (sps), so the maximum seismic data generation rate is 128 kbps.

Seismic data acquisition requires synchronization. In each acquisition operation, the start time is set in the workstation, and all collecting nodes should start acquired data storage simultaneously at the start time. ADCs should also be synchronized by pulse signal. Pulse per second (PPS), provided by LEA-6T, is connected to the collection MCU and ADS1282 for acquisition synchronization, and gateway nodes are also equipped with LEA-6T. Before each acquisition, the workstation sends the acquisition parameters, including the start time, to the gateway nodes; each gateway node sends the acquisition configuration parameters to the collecting nodes, so ADCs are configured and collecting nodes are ready to start acquisition; each gateway node sends an acquisition start instruction to the collecting nodes, according to the current time and PPS falling edge; when PPS is still at a low level, every collecting node receives the instruction, and the collection MCU orders ADC to start seismic signal conversion; when each collection MCU captures the same PPS rising edge, acquired data storage starts. ADC synchronization is done in ADS1282 with the PPS input.

**Figure 3:**
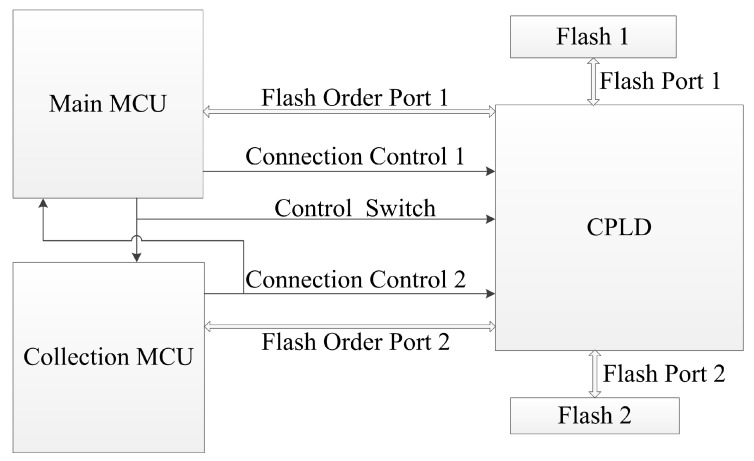
Detailed port connections for flashes.

To achieve quasi real-time seismic data transmission and large capacity local storage simultaneously during the acquisition, the alternate usage of dual flashes is proposed. A complex programmable logic device (CPLD) is used in the alternate usage. Figure 3 shows the detailed port connections for flashes.

As shown in Figure 3, the two flashes are connected to the CPLD. The NAND flash requires 14 pins to fulfill its functions, so each flash port uses 14 pins. Each flash order port also uses 14 pins to connect with a flash port. The connection relationship for flash ports is decided by CPLD according to 3 pins—Control Switch, Connection Control 1 and Connection Control 2 (see Table 1).

As shown in Table 1, the Control Switch decides whether Connection Control 1 or Connection Control 2 determines the connection relationship. In the operations, except seismic acquisition, only the main MCU should use the flashes for stored seismic data transmission, so the Control Switch is set to high by default. Connection Control 1 and Connection Control 2 are also set to high by default. 

For quasi real-time transmission during seismic acquisition, each MCU should connect to the flashes in turn. In the transmission, each seismic data packet contains 1000 bytes of seismic data, which is 250 sampling points in 32-bit sampling. Two seismic data packets involve one page in the NAND flash. The proposed packet format is shown in Table 2.

As shown in Table 2, the total length of a seismic data packet is 1012 bytes. The packet type is used for the identification of different packet types, the storage mark is designed for the system to receive designated packets, and the CRC code is used to catch out the transmission error. Acquisition data volume is calculated using the acquisition configuration parameters, the sampling rate and the acquisition duration. Based on the calculated data volume, the number of data packets is calculated before the acquisition, so the storage marks are also determined. When the main MCU receives the acquisition start instruction, the Control Switch is set to low, so two flash ports connect to two flash order ports, and the collection MCU decides the connection relationship during the acquisition. For acquiring and storing every 4000 bytes of data, the collection MCU changes the electrical level of Connection Control 2, so the connection relationship changes. As Connection Control 2 is also connected to the main MCU, the main MCU notices such changes. When the collection MCU stores the further data in one flash, the main MCU reads the formerly stored data from the other flash and transmits the data. In the collecting node, the seismic data transmission has a delay in its acquisition. The delay is mainly the duration in acquiring the first 4000 bytes. For 4000 sps 32-bit sampling acquisition, such a duration is 0.25 s. When the count for sampling in the collection MCU reaches the calculated data volume, the collection MCU stops storing and informs the main MCU of the end of acquisition. Meanwhile, the main MCU may be in the progress of sending data. The main MCU sets the Control Switch to high after the progress of sending data is finished, and then transmits the remaining acquired seismic data. Failed packets are read and sent based on the storage marks.

With the above design, a collecting node can achieve node positioning, synchronous seismic data acquisition, seismic data storage, and Wi-Fi based short-distance high-rate communication. The proposed alternate usage of dual flashes makes the collecting nodes able to send seismic data in quasi real-time during field operations. Seismic data local storage using NAND flashes can prevent data loss in extreme cases.

### 3.2. Gateway Node

**Figure 4:**
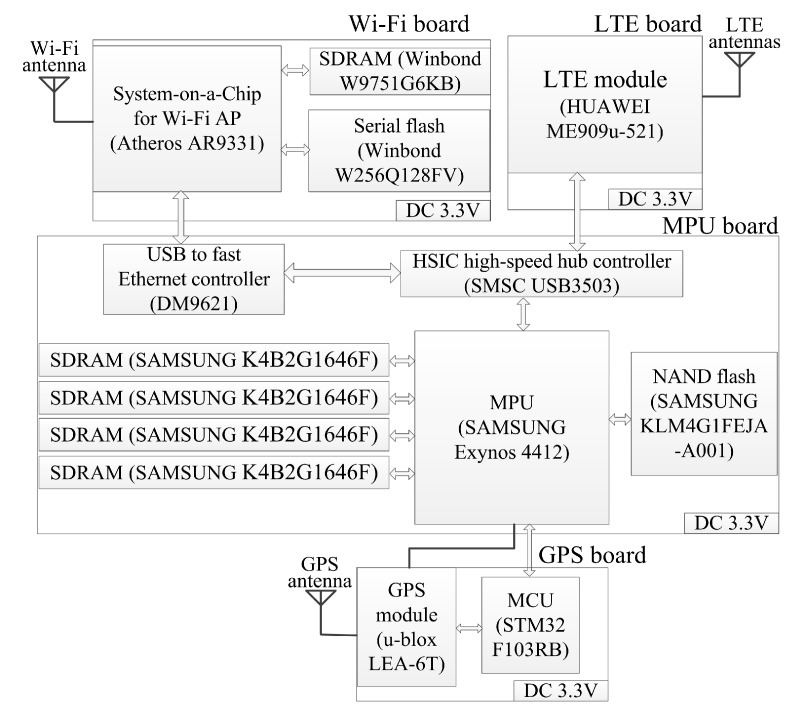
Schematic overview of gateway node structure.

The gateway node should achieve functions including Wi-Fi network management and LTE communication. The gateway node is mainly composed of four boards. Figure 4 shows the schematic overview of the gateway node structure.

The microprocessor unit (MPU) board is the core of the gateway node, deciding the information flow directions inside the node. The Wi-Fi board is mainly designed as a Wi-Fi AP for communication with collecting nodes. The LTE board is designed for communication with the workstation. The global positioning system (GPS) board provides the time reference and PPS for acquisition synchronization, as mentioned in Section 3.1. 

On the MPU board, SDRAMs and a NAND flash are used to support the booting and running of a Linux-based embedded operating system for Exynos 4412. The MPU board connects to the LTE board by USB port and connects to the Wi-Fi board using a fast Ethernet LAN port, so USB3503 and DM9621 are used.

AR9331 supports 802.11b/g/n, fitting with GS2011MIES for a 20 MHz channel in 802.11n 1x1 single stream mode. The SDRAM and flash are used to support the booting and running of a Linux-based embedded operating system for AR9331. An omnidirectional circular rod Wi-Fi antenna is used. The antenna length is less than 0.6 m, and the gain is between 5 dBi and 12 dBi in different circumstances. 

ME909u-521 also runs a Linux-based embedded operating system. ME909u-521 works in LTE frequency division duplexing (LTE FDD), with a peak rate of 50 Mbps for uplink and 100 Mbps for downlink. A main antenna and an auxiliary antenna are used. Each antenna has a length of less than 0.3 m and a gain of no more than 2.5 dBi. 

**Figure 5:**
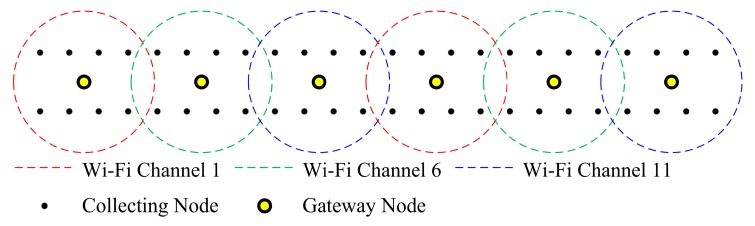
Coverage distribution of Wi-Fi access points (Aps).

The gateway node plays a key role in the network establishment of the system. The upper layer network is initially established when gateway nodes join the LTE network. As there are multiple subnetworks in the array, co-channel interference in Wi-Fi communication should be considered. To deal with co-channel interference, the three non-overlapping channels (Channel 1, Channel 6 and Channel 11) in 2.4 GHz Wi-Fi communication are used. The default channel for each AP is predefined, as gateway nodes are numbered and the default relative position for a gateway node in the array is predetermined. The configuration of the Wi-Fi AP can be changed in real-time, including the channel and Wi-Fi baseband gain. The workstation sends instructions to each gateway node to set the default Wi-Fi baseband gain, considering the operation environment, to achieve high-rate transmission in the subnetwork and low interference with nearby subnetworks. Figure 5 shows an example of the coverage distribution of the Wi-Fi APs, with the array based on the structure in Figure 1.

As shown in Figure 5, the coverage distribution is designed to reduce co-channel interference. The collecting nodes start transmission control protocol (TCP) connections when the service set identifiers (SSIDs) of the gateway nodes are detected. Some collecting nodes can detect two or more of the SSIDs of the gateway nodes. A collecting node can choose a gateway node to connect with according to its received signal strength indication (RSSI) values. When all collecting nodes finish their associations with gateway nodes, subnetworks are formed, so the system can operate the seismic exploration functions.

During field operations, a gateway node is the connecting link between the collecting nodes and the workstation. In the uplink, data packets from collecting nodes should be forwarded to the workstation, so transparent transmission is used; for gateway nodes, data packets, including a status report, are sent to the workstation using LTE. In the downlink, a gateway node receives instructions from the workstation from an LTE module. The MPU receives data packets from the LTE module, parses the packets, analyzes the instructions and decides the following actions. For subnetwork status reports and AP configuration changes, the actions do not involve instructions in the subnetwork. For differential positioning, the received packet is sent to the collecting nodes, to make each collecting node receive the instruction and finish the differential positioning with a high-precision GPS message in the packet. For the seismic data acquisition instruction, a gateway node receives the seismic data acquisition parameters, including start time, end time, sampling rate, digit number, gain of programmable gain amplifier (PGA) and configuration of digital filters; the acquisition duration is calculated using the start and end time; the acquisition duration and configuration parameters of ADC are sent to the collecting nodes in the acquisition configuration instruction; the MPU checks the current time, and sends the acquisition start instruction to the collecting nodes when the MPU captures the PPS falling edge right before the start time; based on the above steps of the gateway node, the collecting nodes can execute the synchronous acquisition mentioned in Section 3.1. 

The maximum seismic data generation rate of each proposed collecting node is 128 kbps in 4000 sps 32-bit sampling. Considering the performance of the adopted modules in the nodes and the proposed operating modes, the system can fulfill the requirement of quasi real-time seismic data transmission in most conditions.

## 4. System Reliability

Exceptions may occur in some extreme conditions. The first aim of the reliability mechanisms is the integrity of the sensor array in order to keep all the collecting nodes maintaining basic functions in the system. 

Most nodes in the system are collecting nodes, so most of the exceptions may occur in the collecting nodes. For a collecting node, the exceptions include acquisition breakdown, data storage failure, GPS signal interruption and Wi-Fi disconnection. Acquisition breakdown, data storage failure and GPS signal interruption are independent from Wi-Fi communication. Acquisition breakdown is due to problems with ADC or the geophone, and it can be detected in an ADC self-test or at the start of acquisition. As there are two NAND flashes in a collecting node, data storage failure includes the breakdown of both flashes and the breakdown of only one flash. The breakdown of both flashes makes seismic data local storage invalid. For breakdown of one flash, quasi real-time seismic data transmission is invalid as the proposed alternative usage requires dual flashes, but other functions, including positioning, Wi-Fi communication, seismic data acquisition and local storage can still be achieved. GPS signal interruption can lead to PPS loss and GPS position failure, caused by the breakdown of the GPS module or special factors, such as extreme weather, and it is possible to resume without manual intervention. Wi-Fi disconnection can be caused by different problems, and a collecting node can notice the disconnection via the disassociation event message of the Wi-Fi module.

**Figure 6:**
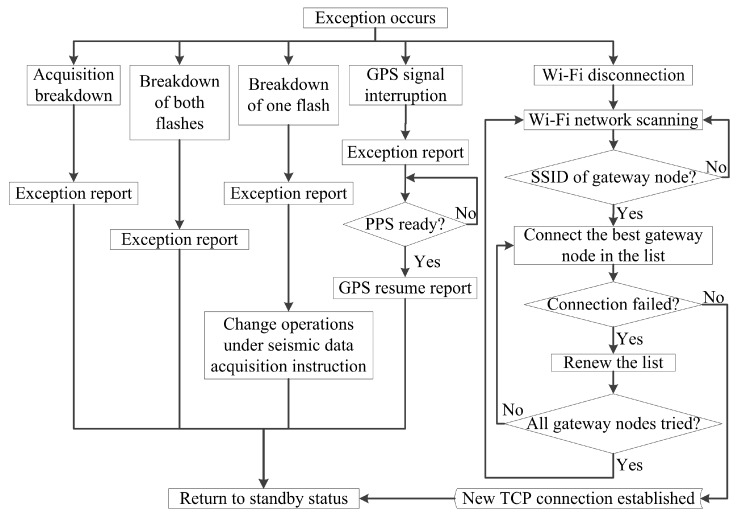
Workflow of collecting node reliability mechanisms.

The proposed subnetwork is a star network, so the failure of one collecting node has no negative impact on the other collecting nodes, and this is an obvious advantage over the multi-hop network structures in terms of system reliability. For collecting node exceptions, the main operations of reliability mechanisms are done in the collecting node, and the workflow of collecting node reliability mechanisms is shown in Figure 6.

As shown in Figure 6, when an exception independent from Wi-Fi communication happens, the node sends an exception report. In the event of acquisition breakdown or the breakdown of both flashes, manual repair is required; after the exception report, the gateway node is informed by the workstation to stop sending instructions to the node. In the event of the breakdown of one flash, the node can still achieve seismic data acquisition and local storage with the remaining flash, and seismic data transmission is done as a relatively independent procedure after the acquisition. Under such circumstances, the collection MCU only strobes the remaining flash during seismic data acquisition, and the main MCU only strobes the remaining flash in data transmission. For GPS signal interruption, the gateway node is informed by the workstation to stop sending instructions to the node after the exception report; the node starts to monitor the condition of the PPS, and operates a resume report when the interruption is relieved; the workstation orders the gateway node to resume sending instructions to the node when it receives the resume report, so the node can resume working. The operator should consider manual intervention if GPS signal interruption persists. For Wi-Fi disconnection, the gateway node sends a Wi-Fi disconnection exception report to the workstation. The mechanism of the collecting node is to establish a new connection with a gateway node. During the establishment of a new connection, the list shows all scanned gateway nodes, and one is removed from the list if the connection has failed; the best gateway node means the one with highest RSSI in the list.

Failure of a gateway node can lead to failure of a subnetwork, so the reliability mechanism for gateway node exception is also designed. For any gateway node exception, all connections fail when the exception happens, or the gateway node can cut off the remaining connections. The workstation and the relevant collecting nodes can notice the disconnections. The workstation orders the nearby gateway nodes to enhance the Wi-Fi baseband gains, so the relevant collecting nodes can detect the SSIDs and establish new connections, following the reliability mechanism for Wi-Fi disconnection. It is most probable for a relevant collecting node to establish the new connection with the nearest gateway node, so the collecting nodes join the one or two remaining nearby subnetworks, and other subnetworks have no change in their connections.

With the above reliability mechanisms, the exceptions in the sensor array can be promptly noticed in the workstation. Some exceptions can be relieved without manual intervention, so the integrity of the collecting node array can be maintained.

## 5. Experiment Results

To verify the feasibility of the proposed system architecture, the experiment was carried out to test the proposed system in data transmission efficiency. 

**Figure 7:**
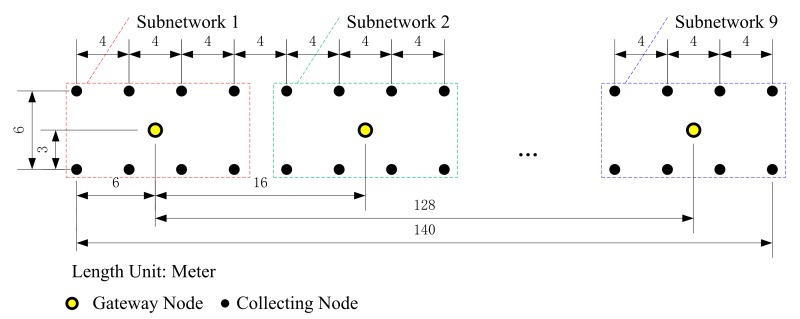
Arrangement of the sensor array.

In the experiment, nine subnetworks were formed. Each subnetwork was composed of a gateway node and eight collecting nodes. The nodes were placed over a rectangular lattice on a closed asphalt road. Due to the limitations of the asphalt road, the in-line offset was 4 m, and the cross-line offset was 6 m. A truck equipped with an air-driven mechanic artificial seismic source was used. Since the usage of a private LTE base station in the city was not authorized, the mobile operator’s LTE network (CMNET) was used in the experiment instead. A remote online terminal with a particular Internet protocol (IP) address on the Internet played the role of the seismic workstation. Figure 7 shows the arrangement of the sensor array in the experiment.

In Figure 7, Subnetwork 1, Subnetwork 2 and Subnetwork 9 are shown in detail. For brevity and clarity, the other subnetworks are replaced by an ellipsis in Figure 7 and each of them had a similar structure to each shown subnetwork. The collecting nodes formed two lines and each line had a length of 140 m. Each gateway node was placed in the center of each subnetwork, and the gateway nodes formed a line with a length of 128 m. In each data acquisition and transmission test operation, all collecting nodes were ordered to execute synchronous 4000 sps 32-bit sampling data acquisition, and the collecting time period was set to 60 s. One shot of the seismic source was executed in each test operation. Ten test operations were done in the experiment. 

**Figure 8:**
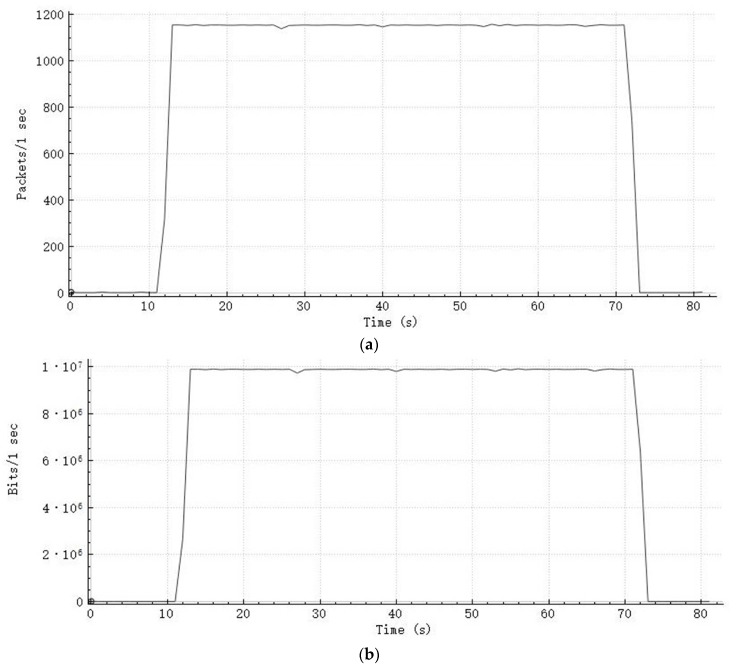
Monitored data transmission condition: (**a**) received packet count, (**b**) received bit count.

Since 72 collecting nodes operated 4000 sps 32-bit sampling, the total seismic data generation rate is 9.216 Mbps. The theoretical seismic data volume in each test operation is 69,120,000 bytes. As each data packet contains 1000 bytes of seismic data, 1152 data packets should be sent per second, theoretically. Packet capture software was used in the online terminal to record the transmission condition. As the terminal received data based on TCP/IP protocol, each captured data packet contains an extra header of 54 bytes with it. Figure 8 shows the monitored data transmission condition of one test operation.

As shown in Figure 8a, the number of received packets per second is around 1152 during the transmission period. For a data acquisition of 60 s, the transmission costs 60.3 s in total. The extra 0.3 s is mainly due to the difference between the communication conditions of the nodes, as some collecting nodes finished the transmissions a little bit later than most of the collecting nodes. As each captured data packet contains extra bytes, the data rate shown in Figure 8b is higher than 9.216 Mbps.

**Figure 9:**
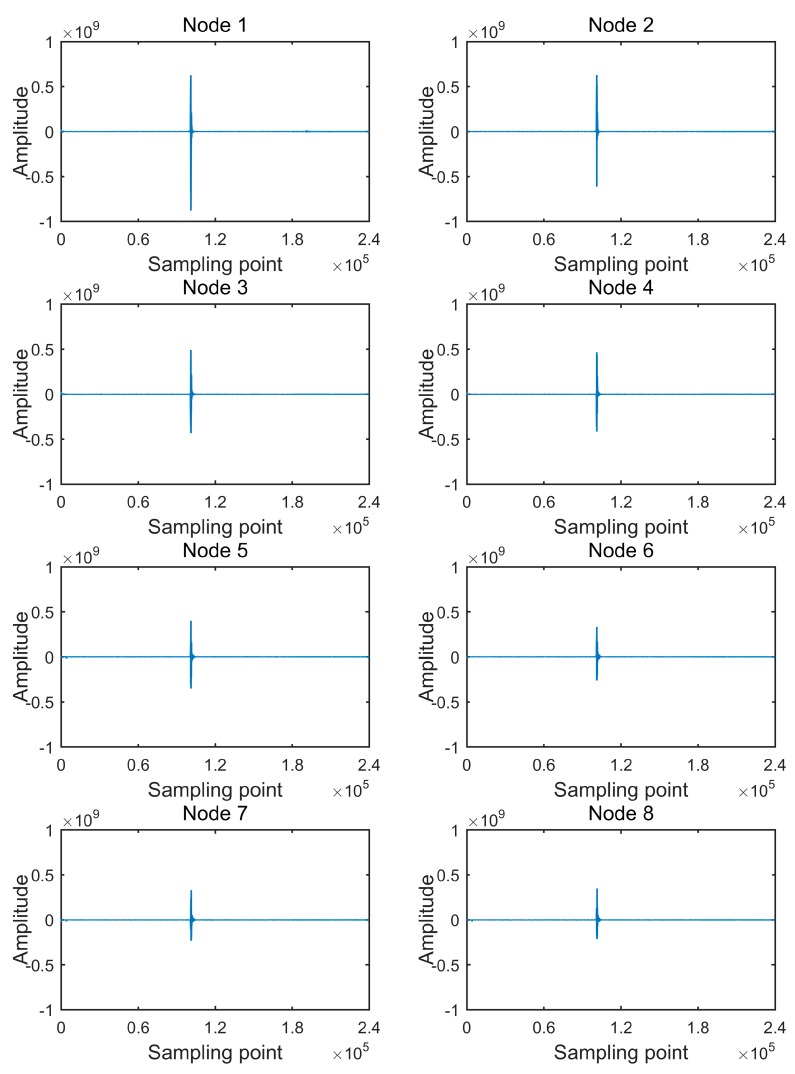
Waveforms of received data.

The received data packets were unpacked and the terminal stored the raw acquired seismic data in the database. The database showed that 69,120,000 bytes were stored for the above test operation, which means no packet loss during the transmission. Figure 9 shows the waveforms of the received seismic data from eight collecting nodes in a subnetwork of the test operation.

**Figure 10:**
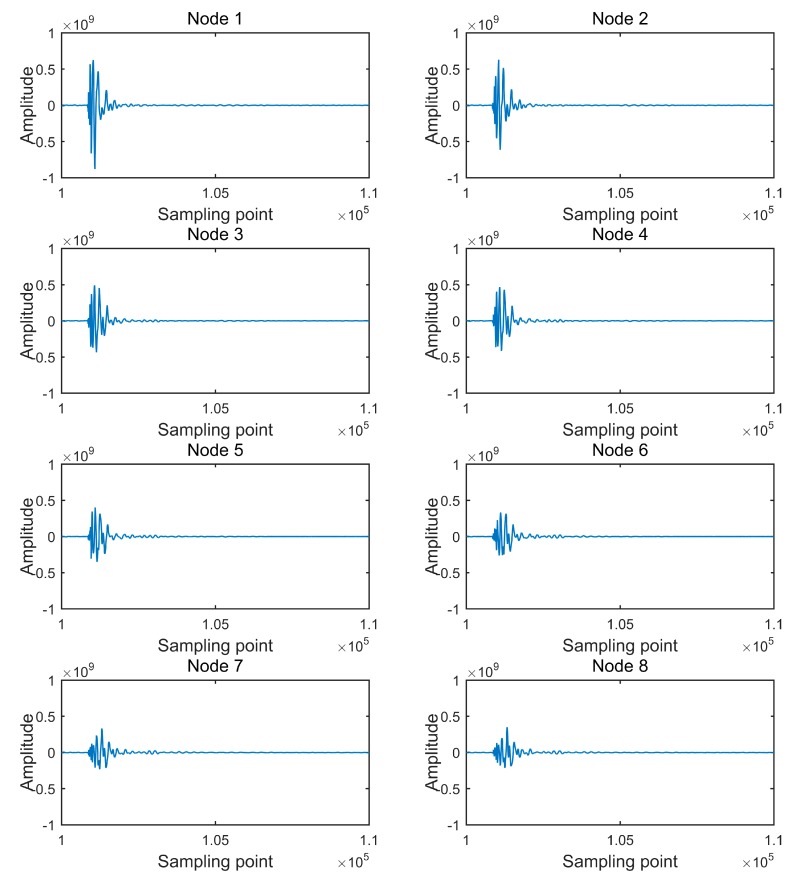
Detailed direct waves of received data.

In Figure 9, “Amplitude” is the 32-bit sampled code value. Each waveform is composed of 240,000 sampling points, which is the complete number of sampling points of a collecting node for a 4000 sps 32-bit sampling acquisition of 60 s. The direct wave of each waveform is between the 100,000th sampling point and the 110,000th sampling point, and detailed direct waves are shown in Figure 10.

The data transmission time consumption records and packet loss rate records of the 10 test operations are shown in Table 3. 

As shown in Table 3, no packet loss happened. Each data transmission time consumption value is between 60.3 to 60.6 s. The fluctuations in network performance could lead to the variations in the data packet delays, so differences appeared in the data transmission time consumptions for test operations with the same parameters. The results show that for 4000 sps 32-bit sampling acquisitions of 60 s, each data transmission time consumption is 0.3 to 0.6 s longer than the acquisition time period. As mentioned in Section 3.1, the data transmission also has a 0.25 s delay in the seismic data acquisition in the collecting node, due to the proposed alternate usage of dual flashes. Considering the above time consumption results and delay in the collecting node, the total delay is short when compared to the total time period, so the above described remote data transmission can be considered quasi real-time for a seismic exploration wireless sensor system.

## 6. Conclusions

For seismic exploration wireless sensor systems, present data transmission systems can hardly achieve a high rate, long distance and high reliability in data transmission simultaneously. A seismic exploration wireless sensor system based on Wi-Fi and LTE is put forward in this paper. The collecting node and gateway node are designed with short antennas, requiring low labor cost for the arrangement of the sensor array. The dual-layer network using LTE for long-distance communication and Wi-Fi for short-distance communication achieves long-distance high-rate seismic data transmission with high reliability. With the proposed nodes and operating modes, quasi real-time seismic data transmission is achieved. Reliability mechanisms are designed to deal with the exceptions. Based on the transmission performance, the proposed system can achieve field operations with high efficiency in seismic exploration.

## Figures and Tables

**Table 1:** Connection relationship for flash ports.

Control Switch	Connection Control 1	Connection Control 2	Flash Port 1	Flash Port 2
High	High	Not concerned	Connected to Flash Order Port 1	High impedance
High	Low	Not concerned	High impedance	Connected to Flash Order Port 1
Low	Not concerned	High	Connected to Flash Order Port 1	Connected to Flash Order Port 2
Low	Not concerned	Low	Connected to Flash Order Port 2	Connected to Flash Order Port 1

**Table 2:** Seismic data packet format

Packet Type	Packet Length	Node Number	Storage Mark	Seismic Data	CRC Code
1 byte	2 bytes	2 bytes	5 bytes	1000 bytes	2 bytes

**Table 3:** Records of 10 test operations.

Operation Number	Data Transmission Time Consumption (s)	Packet Loss Rate (%)
1	60.3	0
2	60.3	0
3	60.4	0
4	60.3	0
5	60.3	0
6	60.4	0
7	60.3	0
8	60.5	0
9	60.6	0
10	60.3	0
